# Supplementary figures and images for: Replication study: androgen receptor splice variants determine taxane sensitivity in prostate cancer
Source: PeerJ. 2018 Apr 16;6:e4661. doi: 10.7717/peerj.4661 (PMC5907780; doi:10.7717/peerj.4661)

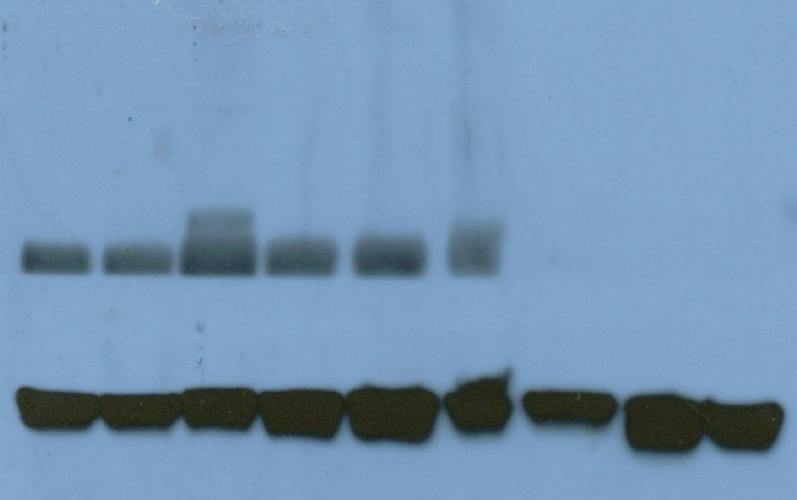

Supplement: Supplemental Information 1 [file peerj-06-4661-s001.png]

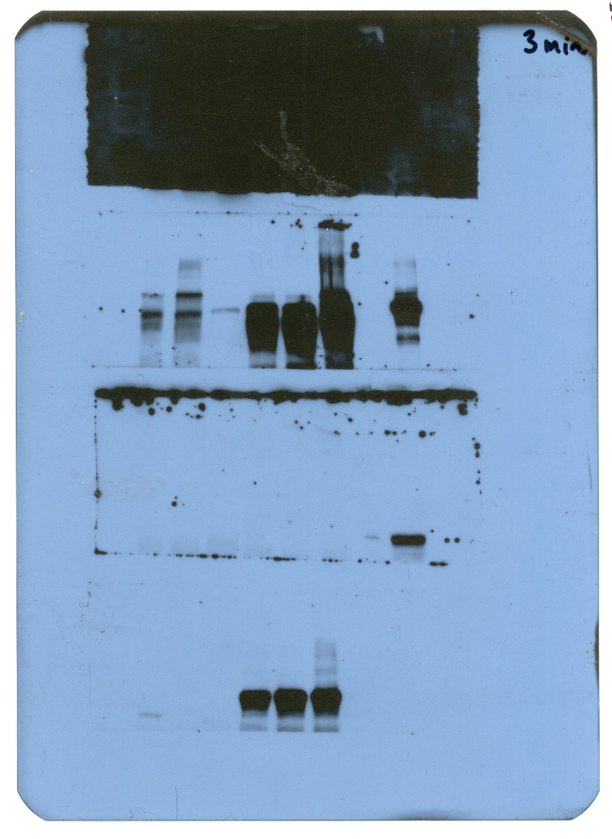

Supplement: Supplemental Information 2 [file peerj-06-4661-s002.png]

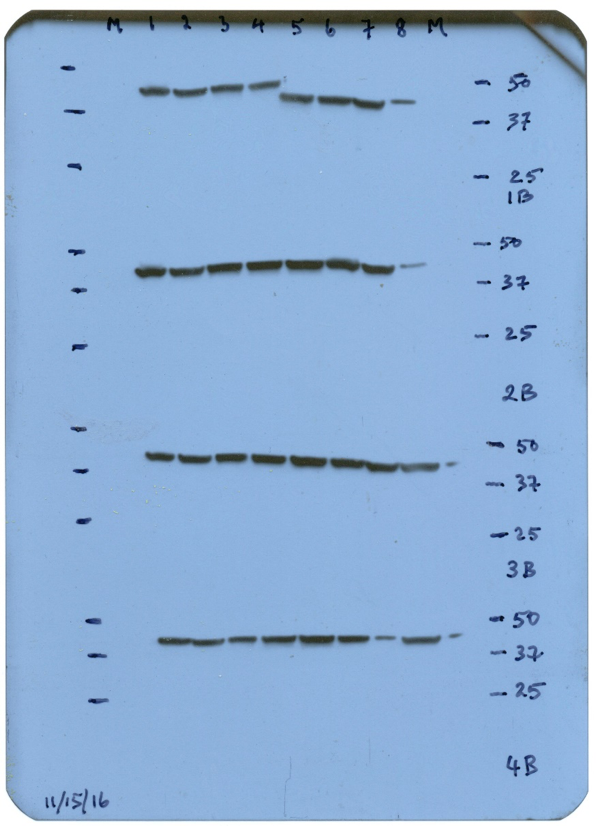

Supplement: Supplemental Information 3 [file peerj-06-4661-s003.png]

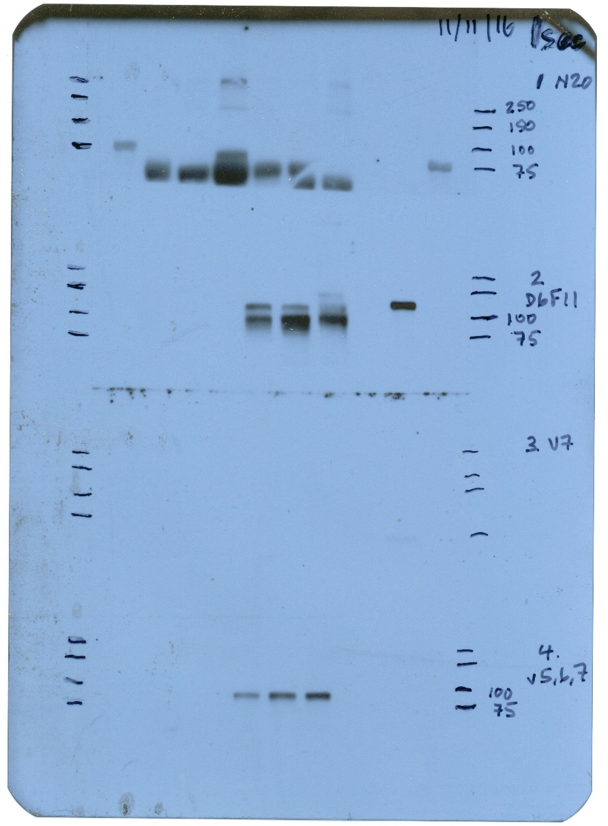

Supplement: Supplemental Information 4 [file peerj-06-4661-s004.png]
